# Glances at the Hospitals

**Published:** 1902-10-18

**Authors:** 


### GLANCES AT THE HOSPITALS.

### CARDIFF INFIRMARY.

The total income for the year was £9,701, being an increase on the previous year of £1,896; and the ordinary expenditure was £8,942, which shows a financial state creditable to all concerned in the management; but while the infirmary has grown considerably the annual income has not kept pace with its growth, and the board of management hopes that the public will increase the income of the institution by subscriptions and donations. This is one of the few English hospitals which publishes neither medical nor surgical statistics. Whether the omission is due to the board of management or to the resident medical staff we cannot say, but it is to be hoped that these statistics will be supplied in the next report. The in-patients numbered 1,663, and of these 884 were surgical, 289 medical, 393 ophthalmic, and 97 gynæcological. Of the total number 798 were discharged cured, 503 relieved or transferred, 132 left for various reasons, 105 died, and 125 remained in the hospital. These numbers show that much interesting matter might be published. The daily cost for provisions per head, including both patients and staff, was 8½d.

### THE LIVERPOOL ROYAL INFIRMARY.

The ordinary income for the past year was £11,789, and the expenses amounted to £14,544. There was a debt against the institution of £3,589. Legacies to the amount of £7,305 were received, but of this sum the larger portion has been invested. This is a questionable proceeding in view of a large adverse balance, unless there are fair hopes of being able to wipe off the latter from next year's ordinary income. We failed to find any statement of the cost per patient, or of the cost per bed occupied. At the beginning of the year there were 259 patients in residence, and 3,218 were admitted during it, making a total of 3,477. Of these 2,905 were discharged, 301 died, and 271 remained under treatment. The average number resident daily was 255, the average duration of residence was 26 days, and the death-rate was 9·37 per cent., but if those dying within 24 hours of admission be excluded, the percentage falls to 7·85. The medical and surgical tables have been prepared with care, and they convey much information, but more details might have been given with advantage. There were 119 cases of hernia. Of these 98 were cured, 11 relieved, 1 transferred, and 9 died. The operation for diseases of the appendix was performed 33 times, 27 cases being cured and 6 dying. Among the major operations we notice that 24 amputations were performed, that 18 were cured, 3 relieved and 3 died; but some remarks concerning these and other operations would have been desirable. The omission is the more remarkable inasmuch as a column of remarks is given in the table of gynæcological operations. The Roentgen rays were applied 326 times. There is no anæsthetic table. It must be noted, however, that in addition to the usual statistics, further details with pathological reports are issued in a separate book form for the use of the medical profession.

### ROYAL ALEXANDRA INFIRMARY, PAISLEY.

The report previous to the one now under notice stated that there was an adverse balance of £836. Mr. Stewart Clarke gave £100 as a special donation to help to wipe off the debt. This good example was followed by Mr. James Coats junior, who presented the infirmary with the balance, so that the infirmary started the year free from debt. The total income for the year, excluding the above-named special donations, was £4,945. The expenditure was £6,698, showing a deficit on the year's working of £1,753. It is, therefore, not without reason that the managers say the question of maintenance is a most serious one, and they appeal to that large class who do not subscribe to come forward in aid of the present subscribers. This sum of £1,753 is not the only debt which must be met sooner or later, for £12,000 still remain unpaid on the building account. There is, however, a silver lining to the Paisley cloud, for £2,000 was received in three donations for the endowment fund of the institution. During the year 780 patients were admitted; 628 were discharged cured or relieved, 62 were dismissed, and 60 died. The average death-rate was 7·8 per cent., but 20 patients died within 48 hours of admission. The average residence of each patient was 39 days. This is unusually high, and if reduced to something like 25 days, would of itself greatly reduce the expenditure. The average daily number resident was 87, the greatest number in the house on any day was 109, and 97 patients were under treatment at the end of the year. The medical and surgical tables are better prepared than is the case in many hospitals. There were 18 cases of acute rheumatism and all recovered. The same may be said of the 25 cases of pneumonia, and also of 18 cases of acute bronchitis. There were 25 amputations, of which 22 were successful. No anæsthetic table is given, and we regret the omission.

### THE BRISBANE HOSPITAL, QUEENSLAND.

THE total ordinary receipts amounted to £14,583, and the total ordinary expenditure to £15,301. Of the receipts only £303 was received from the workpeople themselves. These contributions show a decrease on the previous year, and we can only echo the words of the managers when they say that such a decline passes human understanding. When the people for whose benefit the hospital exists refuse to contribute adequately to its support, it would not be surprising to find that the richer people would withdraw some of their subscriptions and necessitate the curtailment of the charity. While we should deplore such a result, we could not help recognising it as an instance of poetic justice; and we trust that the working men of Brisbane will make some little struggle to make such a thing impossible. The total number of beds in the hospital is 254, and the cubic space per bed is a little over 1,300 feet. At the beginning of the year 190 patients were in residence, and 3,051 were admitted during it, making a total of 3,241 under treatment. Of these 2,584 were discharged cured or relieved, 183 were discharged for various reasons, 15 were transferred to the lunatic asylum, and 277 died. There remained at the close of the year 182. The highest number under treatment at any time was 242; the lowest was 161, and the average number resident was 202. The average residence in the hospital was 21 days. The cost per bed occupied was a fraction over £71 10s., and was made up as follows:—Provisions £21 0s. 8d., surgery, £9 2s. 1d., domestic £10 8s. 9d., establishment charges £2 19s. 2d., rent 11s. 8d., salaries and wages £21 17s., miscellaneous £1 5s., and management £4 5s. 8d. The medical and surgical statistics are clearly arranged and might advantageously be copied by some hospitals in the Old Country. There were 151 cases of typhoid fever with 14 deaths, 13 cases of scarlet fever with 1 death, 147 cases of alcoholism and delirium tremens with 10 deaths, 31 cases of appendicitis with 2 deaths, 35 cases of herniotomy with 3 deaths, 7 cases of ovariotomy with 2 deaths. The out-patients numbered 6,245 and entailed 23,551 attendances. There are 18 physicians or surgeons on the honorary staff, and the resident staff consists of a medical superintendent and three assistants.

